# Age Modifies the Interactive Effect of Loneliness and Social Support on Financial Exploitation Vulnerability in Older Adults

**DOI:** 10.3390/bs14090830

**Published:** 2024-09-17

**Authors:** Gali H. Weissberger

**Affiliations:** Department of Social and Health Sciences, Bar-Ilan University, Ramat Gan 5290002, Israel; gali.weissberger@biu.ac.il

**Keywords:** financial exploitation, older adults, loneliness, social support

## Abstract

Social support and loneliness have been identified as important correlates of financial exploitation vulnerability (FEV) in older adults. However, the potential combined effect of these social factors on FEV remains unclear. Moreover, given that social support and loneliness may become more important as age increases, age may have a moderating effect on the loneliness-social support interaction. Participants were 342 community-living Israeli older adults aged 60 or over (*M* age = 73.37, *SD* = 7.82, 69.1% female) who responded to questionnaires assessing FEV (Financial Exploitation Vulnerability Scale), perceived social support (The Multidimensional Scale of Perceived Social Support), loneliness (UCLA Three-item Loneliness Scale), and sociodemographic characteristics. Two hierarchical linear regression models covarying for demographic variables examined study hypotheses. In a first model, a significant interaction between social support and loneliness was discovered such that high levels of perceived social support attenuated the positive loneliness–FEV association. In a second model, a significant three-way interaction between social support, loneliness, and age was discovered. Probing the interaction revealed that the attenuating effect of social support on the loneliness–FEV link increased with increasing age. Findings suggest that effects of social factors on FEV may interact with each other and with age to predict FEV, and provide insights into when social support may be most relevant in mitigating FEV.

## 1. Introduction

According to the World Health Organization [1], the population of adults over the age of 60 is projected to nearly double from 12% to 22% between the years 2015 and 2050. Experiences of financial exploitation are also likely to increase alongside this steep population increase, consistent with recent trends reported within the United States [2] and underscoring the importance of identifying risk factors of financial exploitation. Financial exploitation is defined as the illegal and/or improper use of an older adult’s funds, property or assets [3]. Aside from significant financial losses incurred from an experience of financial exploitation [4], older adults who experience financial exploitation have been demonstrated to have worse physical, cognitive, and mental health outcomes [5].

Over the recent years, there has been a steady increase in studies that have identified factors associated with increased vulnerability to financial exploitation (or financial exploitation vulnerability, FEV; for review, see [5]). Such risk factors include psychological factors such as depressive and anxiety symptoms (e.g., [6,7]) and physical health factors [8,9,10]. Poor cognitive functioning and/or the presence of underlying dementia conditions have also been demonstrated to increase financial exploitation experiences or FEV [11,12,13], though even older adults without cognitive disorders such as dementia have experienced financial exploitation or evidenced increased FEV (e.g., [12]). Finally, a large body of work has pointed to social factors such as social network size, social support, and loneliness as relevant correlates of FEV (e.g., [6,12,14,15,16]). However, few studies have examined how these social risk factors may interact with one another to predict FEV (for exceptions, see [14,17]).

Perceived social support has been defined as the perception or experience of having the availability of social resources through formal and informal relationships [18]. Conversely, loneliness is an aversive state that occurs when there is a discrepancy between the interpersonal relationships one wishes to have and the current relationships one perceives to have. In other words, it is a feeling that one’s social relationships are inadequate vis-à-vis one’s desired relationships [19]. The feeling of loneliness is subjective, in that two people may have an equal number of friends or social support systems in place yet appraise their adequacy quite differently [19,20]. While enhancing social support is generally thought of as a way to reduce loneliness, certain factors (e.g., urban vs. rural residential status [21]) have been shown to moderate the relationship between perceived social support and loneliness, and a person with high levels of social support may still experience loneliness [21].

Numerous studies have demonstrated an association between lower FEV and higher social support or related concepts [6,12,14,22,23]. For example, a study by Beach and colleagues [14] showed that older adults who reported large social networks but low perceived social support were at the highest risk for financial exploitation. Additionally, in a study by Liu et al. [22] that examined various aspects of social support including positive and negative interactions with close network members, negative interactions were associated with FEV while positive interactions were not. These findings suggest that negative aspects of social interactions are a risk factor for potential FE. In this regard, research has also demonstrated a relationship between loneliness and FEV [15,16,24,25]. For example, Lim et al. [16] showed that interpersonal dysfunction characterized by loneliness and dissatisfaction subsequently predicted increases in FEV over a six-month period.

Various explanations have been proposed for the relationship between social support and loneliness and FEV. With regards to social support, financial exploitation usually involves a social transaction [5] and the lack of close others in emotionally and instrumentally supportive roles may increase the likelihood of bad actors gaining access to an older adult’s funds [26]. Even when older adults report a social network comprised of close family and friends, it is important to note that incidents of financial exploitation most often involve a family member or known other [27,28], thereby underscoring the importance of considering an older adult’s appraisal of his or her social resources or his or her satisfaction with social relationships in this context. Loneliness may also increase FEV by increasing the likelihood that older adults seek connections with others in unconventional ways (e.g., consumer-based social ties; see [29]). Consistent with this, Alves and Wilson [15] demonstrated that loneliness among fraud victims was associated with more money lost and more weekly contacts with telemarketers.

Another mechanism by which loneliness may be associated with FEV is via susceptibility to persuasion [24]. Specifically, in a study by Wen et al. [24], susceptibility to persuasion mediated the relationship between loneliness and fraud vulnerability. The authors argued that lonely individuals are more inclined to agree with others during social interactions, thus increasing their susceptibility to persuasion and subsequent fraud vulnerability [24]. Such findings point to the importance of understanding how social factors may interact together to predict FEV. For example, it may be the case that social support is particularly protective in mitigating FEV for older adults who experience high levels of loneliness.

The effects of social support and loneliness on FEV may become even more pronounced with increasing age. According to socioemotional selectivity theory [30,31], as time horizons decrease with increasing age, social goals that are emotional in nature (e.g., experiencing positive emotional states) are prioritized over knowledge-related social goals (e.g., mastering social and nonsocial skills). Thus, prioritizing quality over quantity of social relationships takes precedence due to this shift in focusing on emotionally-gratifying goals (vs. expansive goals) in light of these perceived time limitations. As a result, social networks become tighter and include more emotionally close relationships [31]. Consistent with this, research has repeatedly demonstrated the vital role of social relationships, and specifically social connectedness, to the well-being of older adults ([32] for review). As time horizons continue to shrink with increasing age, the importance of the quality of social connections is likely to increase given poorer health and increased dependencies that often occur with advancing age. Such age-related changes have also been shown to be related to increased FEV [27,33]. For example, increased dependencies on others is a known risk factor of FEV [33]. As such, having good quality social supports may serve as an even stronger buffer against FEV-related risk factors such as loneliness as one’s age increases.

The present study aimed to examine the interactive effect of social support and loneliness on FEV, and whether age modifies this effect, in a community-based sample of Israeli adults aged 60 or older. In a first aim, the interactive effect of social support and loneliness on FEV was tested. It was hypothesized (H1) that higher levels of social support would mitigate the positive association between loneliness and FEV. In a second aim, the interactive effect of age was added to examine whether the social support by loneliness interaction strengthens with increasing age. It was hypothesized (H2) that the protective effect of social support on the loneliness–FEV link would increase with increasing age.

## 2. Materials and Methods

### 2.1. Participants and Procedure

Participants were recruited using a snowball sampling technique to take part in a study aimed at understanding social factors related to FEV. Specifically, participants within the Israeli community were approached by research assistants from January to April 2024. Inclusion criteria included being over the age of 60, being able to speak and read Hebrew at least at a “very good” level, and having no known/diagnosed cognitive or neurological impairments or conditions. Based on these inclusion criteria, a total of 469 participants were recruited to participate. Of the 469 recruited participants, 342 participants (*M* age = 73.37, *SD* = 7.82, range = 60–99, 69.1% female) were included in the present analyses for responding to 80% or more of the questionnaires and completing all scales relevant to the present study.

Eligible participants agreeing to participate in the study were personally sent a designated Qualtrics link by the research assistant who had made initial contact with them. Participants first provided their informed consent via the Qualtrics link. Those who provided their informed consent continued on to complete the study scales. Personal details were neither required nor requested, thereby guaranteeing anonymity. All scales included in the study were back-translated into Hebrew by two experienced bilingual psychologists.

### 2.2. Instruments

*Sociodemographic variables.* Age (in years) was included as a covariate in the first set of models that examined the interactive effect of social support and loneliness on FEVS. Sex (male = 0, female = 1) and relationship status (in a relationship = 1; not in a relationship = 0) were also included as covariates. Education level, assessed on an eight-point scale from 1 “no formal education” to 8 “doctoral degree” was additionally included as a covariate. Gross combined monthly household income was also assessed and included as a covariate in all models. Response options ranged from 1 (up to 2500 New Israeli Shekels (NIS)) to 11 (over 30,001 New Israeli Shekels (NIS). Finally, participants indicated which diagnoses from a list of 14 medical conditions (e.g., cancer, heart disease) they received from a physician [34]. Sum of illnesses was included as a covariate in all analyses.

*Financial Exploitation Vulnerability.* FEV was assessed using the Financial Exploitation Vulnerability Scale (FEVS; [35]). Participants respond to 17 questions that assess risk for financial exploitation based on the context of an individual’s perceived financial reality including one’s financial awareness, psychological vulnerability, and relationship strain [35]. Example items include, “how likely is it that anyone now wants to take or use your money without your permission?”, “who manages your money day to day?” and “how satisfied are you with this [money management] arrangement?” Response options vary per item. Scores on each individual item are summed, with higher scores indicating greater FEV. Reliability in this study was acceptable (Cronbach’s alpha = 0.80), consistent with previous studies that have utilized the Hebrew version of this measure [23,36]. The scale was also shown to have good sensitivity and specificity in detecting risk of financial exploitation (AUC = 0.834, 95% CI: 0.78–0.89; [35]).

*Perceived social support.* The 12-item Multidimensional Scale of Perceived Social Support [37] was used to measure perceived social support. The scale asks participants to rate their agreement from 1 (“strongly disagree”) to 5 (“strongly agree”) across the 12 items (e.g., “There is a special person who is around when I am in need”, “I get the emotional help and support I need from my family”, “I can count on my friends when things go wrong”). The mean of responses is calculated, with higher scores reflecting higher levels of social support. Due to low inter-item correlation with item 9 of the scale, this item was dropped and mean social support was calculated without this item. Cronbach’s alpha for the 11 items was 0.93. It should be noted that results were not significantly altered when this item was included in the analyses.

*Loneliness.* The UCLA Three-item Loneliness scale was used to measure self-reported loneliness [38]. Participants responded to three items assessing various aspects of the loneliness experience (e.g., lack of companionship) using a rating scale of 0 (“never”) to 4 (“almost always”). Scores are averaged with higher scores reflecting higher levels of loneliness. Reliability in this study was high (Cronbach’s alpha = 0.92).

### 2.3. Statistical Analyses

Analyses were conducted using SPSS 28 software. All variables were examined for normality and outliers, and bivariate associations between all study variables were conducted using Pearson or Spearman correlations. To test hypothesis 1, a hierarchical linear regression model was conducted with FEVS as the outcome variable. The first step included covariates of age, sex, education level, relationship status, income, and sum of illnesses. The second step added main effects of loneliness and social support. The final step added the interaction term for loneliness and social support. To test hypothesis 2, a second hierarchical linear regression model was conducted with the first step including all covariates except age, which was entered in the second step along with social support and loneliness. Step 3 added the three two-way interaction terms of age by loneliness, age by social support, and social support by loneliness. Finally, step 4 added the three-way interaction term of age by social support by loneliness. Variables included as part of an interaction term were centered to the mean in both models. Interaction effects were further probed using Models 1 and 3 of the PROCESS 4.2 Macro for SPSS [39].

A power analysis was conducted for the more robust model. This revealed that the sample size required for detecting a medium effect size of *f*^2^ = 0.15, with power of 0.95 and 12 predictors, was 154 indicating that the current sample was sufficient for examining the study models. Potential multicollinearity between the predicting variables was rejected, as values of tolerance ranged between 0.725 and 0.940, and values of the variance inflation factor (VIF) ranged between 1.064 and 1.460.

## 3. Results

### 3.1. Bivariate Correlations between Study Variables

Means, standard deviations, range, and bivariate Pearson and Spearman correlations between study variables are presented in Table 1. Increased FEV was associated with lower education level (*p* < 0.001), less reported gross household monthly income (*p* < 0.001), more illnesses (*p* < 0.001), lower perceived social support (*p* < 0.001), and increased loneliness (*p* < 0.001). Those in a relationship had lower FEV (*p* = 0.013). There were no differences between men and women in FEV (*p* = 0.672) and age was not correlated with scores on the FEVS (*p* = 0.208).

### 3.2. Hierarchical Linear Regression Model Examining the Interactive Effect of Social Support and Loneliness on FEV

To examine whether social support and loneliness interact to predict FEV, a hierarchical linear regression was conducted. Step 1 (Table 2a) of the model regressed FEV on covariates of age, sex, education level, income, sum of illnesses, and relationship status. This step revealed main effects of education level (*B* = −0.36, *β* = −0.16, *t* = −2.78, *p* = 0.006), income (*B* = −0.27, *β* = −0.17, *t* = −2.84, *p* = 0.005), and sum of illnesses (*B* = 0.77, *β* = 0.25, *t* = 4.75, *p* < 0.001). Main effects of social support and loneliness (Step 2, Table 2b) were also discovered (*B* = −0.80, *β* = −0.15, *t* = −2.84, *p* = 0.005; *B* = 1.51, *β* = 0.35, *t* = 6.74 *p* < 0.001, respectively). Inclusion of these variables explained an additional 17.3% variance in FEVS scores. In support of Hypothesis 1, a significant interaction between social support and loneliness was also found (Step 3, Table 2c; *B* = −0.56, *β* = −0.13, *t* = −2.72, *p* = 0.007), explaining an additional 1.4% of variance in FEVS scores.

**Figure 1:**
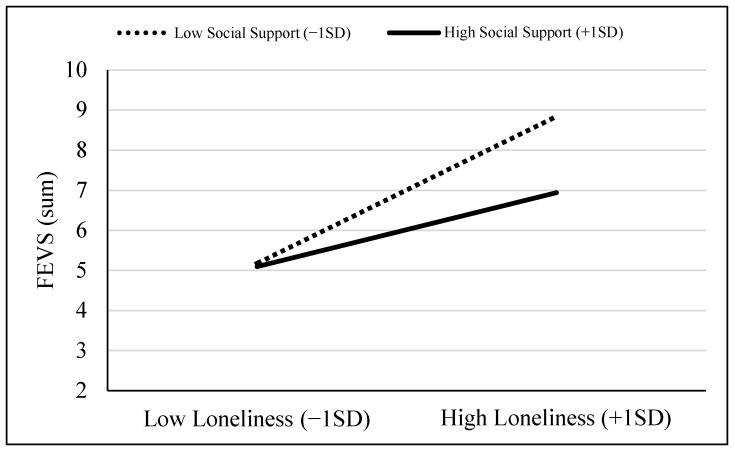
Display of the two-way loneliness by social support interaction.

The interaction was probed further using Model 1 of the SPSS PROCESS Macro [39]. This revealed that the positive effect of loneliness on FEVS weakened as levels of perceived social support increased (Figure 1). Examining the conditional effects of loneliness on FEVS based on different values of social support revealed that among individuals with low levels of perceived social support (scores of −1 standard deviation (*SD*) or below), the effect was strongest (*B* = 1.83, SE = 0.25, *t* = 7.28, *p* < 0.001). Among individuals with high levels of perceived social support (scores of +1*SD* or above), the effect was weakest though still significant (*B* = 0.92, SE = 0.31, *t* = 3.00, *p* = 0.003).

### 3.3. Hierarchical Linear Regression Model Examining Age as a Moderator of the Social Support by Loneliness Interactive Effect on FEV

To examine whether age moderates the loneliness by social support interaction, an additional hierarchical regression analysis was conducted. Covariates of sex, education level, income, sum of illnesses, and relationship status were included in Step 1 (Table 3a). As in the first model, education level (*B* = −0.35, *β* = −0.16, *t* = −2.76, *p* = 0.006), income (*B* = −0.26, *β* = −0.16, *t* = −2.73, *p* = 0.007), and sum of illnesses (*B* = 0.73, *β* = 0.24, *t* = 4.61, *p* < 0.001) were significantly related to FEV. In Step 2 (Table 3b), there was a marginal main effect of age (*B* = −0.05, *β* = −0.09, *t* = −1.92, *p* = 0.056), and main effects of social support (*B* = −0.80, *β* = −0.15, *t* = −2.84, *p* = 0.005) and loneliness (*B* = 1.51, *β* = 0.35, *t* = 6.74, *p* < 0.001). The two-way interactions (age × social support; age × loneliness; social support × loneliness) were then entered into Step 3 (Table 3c). Only the social support by loneliness interaction was significant (*B* = −0.52, *β* = −0.12, *t* = −2.53, *p* = 0.012). Finally, in Step 4, the three-way age by loneliness by social support interaction was significant (Table 3d; *B* = −0.07, *β* = −0.13, *t* = −2.45, *p* = 0.015) explaining an additional 1.1% variance in FEVS scores.

**Figure 2:**
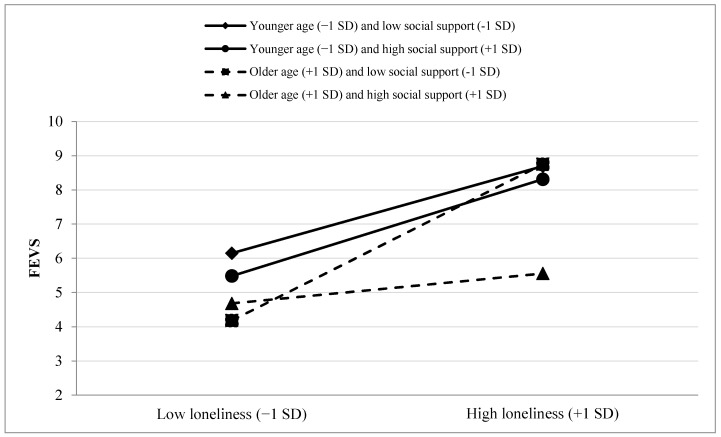
Display of the three-way age by loneliness by social support interaction.

The three-way interaction was further probed using PROCESS. This was done by examining the conditional effect of the loneliness by social support interaction on FEVS scores at three different age groups: participants whose chronological age fell below one standard deviation (*SD*) of the mean (−1*SD*), within one *SD* of the mean, and above one *SD* of the mean (+1*SD*). This revealed that the loneliness by social support interaction effect increased with increasing age. Specifically, for the youngest older adults (−1*SD*), the loneliness by social support interaction effect was not significant (*B* = 0.09, *p* = 0.786). The effect was significant for older adults within 1*SD* of the mean age in the sample (*B* = −0.49, *p* = 0.018) and it was strongest for those above 1*SD* of the mean age in the sample (*B* = −1.13, *p* < 0.001). Further probing of these relationships was done by examining conditional effects of loneliness on FEVS scores at different levels of social support and age groups. In doing so, it was discovered that the effect of loneliness on FEVS scores was significant for all combinations of social support levels (−1*SD*, within 1*SD*, and +1*SD*) and age groups except the oldest older adults who had high levels of social support. In other words, social support appears to have an attenuating effect on the positive association of loneliness and FEV in the oldest older adults. A display of these relationships can be viewed in Figure 2. Table 4 displays the statistical parameters for these conditional effects.

## 4. Discussion

This study examined the interactive role of perceived social support and loneliness on FEV in a community-based sample of Israeli older adults, and whether this interactive effect is moderated by age. In line with the first hypothesis, it was found that the relationship between loneliness and FEV was attenuated at higher levels of perceived social support, suggesting a protective effect of social support on the loneliness–FEV link. In line with the second hypothesis regarding age, it was discovered that the attenuating effect of social support on the loneliness–FEV link was strongest for the oldest older adults in the sample. Specifically, in the oldest older adult participants, the relationship between loneliness and FEV was rendered nonsignificant in those with the highest reported levels of perceived social support.

Findings of this study contribute to existing knowledge regarding the relevance of social factors to FEV. Consistent with previously reported studies [6,12,14,22,23], low perceived social support and high loneliness were individually associated with higher FEV as demonstrated by the significant main effects of each. Additionally, a significant two-way interaction between social support and loneliness implies that the positive effect of loneliness on FEV is attenuated amongst older adults who report high levels of perceived social support. Loneliness may independently increase FEV via various mechanisms, including the seeking of social connections in unconventional ways (e.g., telemarketers, [15]) or increasing one’s susceptibility to persuasion [24]. Considering these two mechanisms in conjunction, lonely individuals may not only seek to form unconventional social ties, thereby increasing their exposure to potentially bad actors, but may also be more persuadable to various marketing tactics used by those with whom they form such ties. However, high levels of perceived social support may modify these types of behaviors as supportive individuals (either emotionally, instrumentally, or both) may be able to detect the nefarious intentions of others and intervene when necessary.

Findings of this study also suggest that social support may serve as a protective factor in mitigating the association between high loneliness and increased FEV, specifically amongst the oldest older adults. This was evidenced by probing the three-way interaction between age, loneliness, and social support, which revealed that the positive association between loneliness and FEV was nullified only for the oldest older participants who reported high levels of social support. According to socioemotional selectivity theory [30,31], the quality of social relationships becomes more important as time horizons shrink. In this context, a study by Weissberger et al. [23] found that social support was associated with FEV only amongst older adults with older subjective ages (i.e., older adults who feel older than their chronological age), suggesting that social support is particularly important for individuals who feel “older” and potentially view their time horizons as less broad. In the context of the present study, strong supportive relationships may therefore become increasingly important in mitigating the loneliness–FEV association amongst the oldest-old whose horizons are (at least objectively) the most constricted.

Although the underlying mechanisms for the relationships investigated in this study were not examined, factors associated with FEV such as dependency on others [33], cognitive impairment [12,40], and physical morbidity [8,9,10] may play a role in the discovered associations. Specifically, many of these FEV risk factors become more prominent as one ages and may thus intensify the need for strong social resources such as social support. Given that loneliness has also been demonstrated to increase risk factors such as cognitive impairment and physical morbidity in older adults [41,42,43], high levels of social support may be even more effective in mitigating the loneliness–FEV link as age increases.

There are limitations to this study that deserve attention. First, the sample was a convenience-based sample of older adults who required access to the internet to complete the online survey. This inevitably reduces the representativeness of the sample and the generalizability of the study findings. Additionally, conclusions regarding directionality of relationships cannot be made given the cross-sectional nature of the study. For example, while loneliness and social support were discussed as factors that can affect FEV, it is possible that increased FEV has a downstream effect on loneliness and social support. A final set of limitations relate to the measurements used in the present study. While perceived social support was examined in this study, utilizing objective markers of social support, and examining subcomponents of social support (e.g., emotional vs. instrumental) may provide unique insights into its role in modifying risk factors of FEV. Additionally, subcomponents of social support (support from friends versus family, formal vs. informal support) may be examined to further elucidate its moderating role in the loneliness–FEV link. Finally, monomethod bias is apparent in the present study. Given that each construct was measured utilizing just one representative questionnaire, examining the study models using other methods and different questionnaires (e.g., history of financial exploitation experiences) will be important in order to broaden study implications.

## 5. Conclusions

Findings of the present study suggest that the role of social support as a protective factor in the loneliness–FEV association is most relevant in the oldest old. These findings have important implications for healthcare professionals, policy makers, and researchers developing and utilizing techniques aimed at reducing FEV and preventing the experience of financial exploitation in older adults. The findings support the notion that promoting social health in older age can be a protective factor against FEV and may serve as an effective preventative tool. Specifically, preventative measures developed to reduce FEV may focus on enhancing social support amongst the oldest old who report high levels of loneliness. On the other hand, directly targeting loneliness in younger older adults may be more effective in reducing FEV, as the moderating effect of social support was less prominent in the youngest older adults in the sample. More generally, findings also highlight the importance of considering interactive relationships between various risk factors and FEV, as associations of FEV with certain risk factors may weaken or strengthen based on the presence of other factors. Such investigations will undoubtedly affect the development and effectiveness of focused interventions and preventative techniques. Future studies may consider longitudinal examinations of the relationships reported in the present investigation as well as examining subcomponents or objective measures of social support to expand upon the reported findings.

## Figures and Tables

**Table 1:** Descriptive information and correlation matrix for study variables.

				Bivariate Pearson or Spearman Correlations ^a^								
Variable		Mean (Range)	*SD*	1	2	3	4	5	6	7	8	9
1	Age	73.37 (60–99)	7.82	1	−0.075	−0.088	−0.210 **	0.233 **	−0.221 **	−0.075	0.154 **	0.068
2	Sex (% female)	69.01%	-		1	−0.022	−0.047	0.086	−0.159 **	0.058	−0.064	−0.023
3	Education level	3.92 (1–8)	1.95			1	0.479 **	−0.086	0.086	0.167 **	−0.224 **	−0.261 **
4	Income	6.18 (1–11)	2.76				1	−0.209 **	0.275 **	0.146 **	−0.202 **	−0.302 **
5	Sum of Illnesses	2.22 (1–8)	1.44					1	−0.158 **	−0.072	0.121 *	0.239 **
6	Relationship status (% in a relationship)	48.38%	-						1	0.123 *	−0.108 *	−0.135 *
7	Perceived social support (mean)	3.81 (1–5)	0.80							1	−0.481 **	−0.372 **
8	Loneliness (mean)	1.07 (0–4)	1.03								1	0.455 **
9	FEVS (sum)	6.83 (0–22)	4.4									1

Notes: FEVS = Financial Exploitation Vulnerability Scale; *SD* = standard deviation. * *p*-value ≤ 0.05; ** *p*-value ≤ 0.01. ^a^ Bivariate associations with education level, sum of illnesses, and loneliness were examined using Spearman correlations.

**Table 2:** Results of the hierarchical linear regression model examining the two-way social support by loneliness interaction on FEVS.

	Predictor	*R*^2^ Change	*B*	*SE*	LLCI ^a^	ULCI ^a^	*β*	*t*	*p*-Value
a. Step 1		0.171 ***							
	Age		−0.034	0.030	−0.093	0.025	−0.060	−1.140	0.255
	Sex ^1^		−0.725	0.485	−1.679	0.230	−0.076	−1.494	0.136
	Education level ^2^		−0.356	0.128	−0.609	−0.104	−0.158	−2.777	0.006
	Income ^3^		−0.272	0.096	−0.460	−0.083	−0.170	−2.839	0.005
	Sum of Illnesses		0.766	0.161	0.448	1.083	0.250	4.746	<0.001
	Relationship status^4^		−0.519	0.482	−1.468	0.430	−0.058	−1.076	0.283
b. Step 2		0.173 ***							
	Social support		−0.797	0.280	−1.348	−0.246	−0.145	−2.844	0.005
	Loneliness		1.507	0.224	1.067	1.947	0.351	6.737	<0.001
c. Step 3		0.014 **							
	Social support × loneliness		−0.556	0.204	−0.957	−0.154	−0.130	−2.723	0.007
	Total *R*^2^	0.358							

Notes: ^1^ 0 = male, 1 = female; ^2^ 1 = no formal education, 2 = partial high school, 3 = full high-school, 4 = partial undergraduate studies, 5 = undergraduate degree/diploma, 6 = partial graduate, 7 = MA/MSc/graduate degree, 8 = doctoral degree; ^3^ Gross combined monthly household income in NIS (0 = no income, 1 = up to 2500, 2 = 2501–4000, 3 = 4001–5000, 4 = 5001–6500, 5 = 6501–8000, 6 = 8001–10,000, 7 = 10,001–13,000, 8 = 13,001–17,000, 9 = 17,001–24,000, 10 = 24,001–30,000, 11 = more than 30,001). ^4^ 0 = not in a relationship; 1 = in a relationship; ^a^ Lower and upper limits for 95% confidence intervals for the unstandardized beta values (*B*). ** = *p* ≤ 0.01; *** = *p* ≤ 0.001.

**Table 3:** Results of the hierarchical linear regression model examining the three-way age by social support by loneliness interaction on FEVS.

	Predictor	*R*^2^ Change	*B*	*SE*	LLCI ^a^	ULCI ^a^	*β*	*t*	*p*-Value
a. Step 1		0.168 ***							
	Sex ^1^		−0.650	0.481	−1.596	0.296	−0.068	−1.351	0.178
	Education level ^2^		−0.354	0.128	−0.607	−0.102	−0.157	−2.761	0.006
	Income ^3^		−0.260	0.095	−0.447	−0.073	−0.163	−2.734	0.007
	Sum of Illnesses		0.730	0.158	0.419	1.042	0.238	4.610	<0.001
	Relationship status ^4^		−0.423	0.475	−1.358	0.512	−0.047	−0.891	0.374
b. Step 2		0.176 ***							
	Age		−0.051	0.027	−0.104	0.001	−0.091	−1.921	0.056
	Social support		−0.797	0.280	−1.348	−0.246	−0.145	−2.844	0.005
	Loneliness		1.507	0.224	1.067	1.947	0.351	6.737	<0.001
c. Step 3		0.022 **							
	Age × social support		−0.058	0.037	−0.130	0.014	−0.081	−1.574	0.116
	Age × loneliness		0.01	0.029	−0.046	0.066	0.018	0.347	0.729
	Social support × loneliness		−0.516	0.210	−0.919	−0.114	−0.121	−2.525	0.012
d. Step 4		0.011 *							
	Age × social support × loneliness		−0.072	0.029	−0.129	−0.014	−0.127	−2.448	0.015
	Total *R*^2^	0.378							

Notes: ^1^ 0 = male, 1 = female; ^2^ 1 = no formal education, 2 = partial high school, 3 = full high-school, 4 = partial undergraduate studies, 5 = undergraduate degree/diploma, 6 = partial graduate, 7 = MA/MSc/graduate degree, 8 = doctoral degree; ^3^ Gross combined monthly household income in NIS (0 = no income, 1 = up to 2500, 2 = 2501–4000, 3 = 4001–5000, 4 = 5001–6500, 5 = 6501–8000, 6 = 8001–10,000, 7 = 10,001–13,000, 8 = 13,001–17,000, 9 = 17,001–24,000, 10 = 24,001–30,000, 11 = more than 30,001). ^4^ 0 = not in a relationship; 1 = in a relationship; ^a^ Lower and upper limits for 95% confidence intervals for the unstandardized beta values (*B*). * = *p* ≤ 0.05; ** = *p* ≤ 0.01; *** = *p* ≤ 0.001.

**Table 4:** The conditional effects (*B*) of loneliness on FEVS for different levels of social support and age groups.

Social Support Level	Age Group	*B*	*SE*	*t*	*p*-Value
Social support −1*SD*	Age −1*SD*	1.272	0.392	3.2440	0.001
Social support −1*SD*	Age within 1*SD*	1.748	0.252	6.9310	<0.001
Social support −1*SD*	Age +1*SD*	2.285	0.386	5.9230	<0.001
Social support within 1*SD*	Age −1*SD*	1.351	0.349	3.8760	<0.001
Social support within 1*SD*	Age within 1*SD*	1.307	0.231	5.6600	<0.001
Social support within 1*SD*	Age +1*SD*	1.258	0.326	3.8610	<0.001
Social support +1*SD*	Age −1*SD*	1.414	0.466	3.0350	0.003
Social support +1*SD*	Age within 1*SD*	0.954	0.307	3.1060	0.002
Social support +1*SD*	Age +1*SD*	0.437	0.443	0.9870	0.325

Note: *SD* = standard deviation; *SE* = standard error.

## Data Availability

Data can be made available upon request to the study author.
